# Nanopore Sequencing Is a Credible Alternative to Recover Complete Genomes of Geminiviruses

**DOI:** 10.3390/microorganisms9050903

**Published:** 2021-04-23

**Authors:** Selim Ben Chehida, Denis Filloux, Emmanuel Fernandez, Oumaima Moubset, Murielle Hoareau, Charlotte Julian, Laurence Blondin, Jean-Michel Lett, Philippe Roumagnac, Pierre Lefeuvre

**Affiliations:** 1 CIRAD, UMR PVBMT, F-97410 St Pierre, La Réunion, France; selim.ben_chehida@cirad.fr (S.B.C.); murielle.hoareau@cirad.fr (M.H.); jean-michel.lett@cirad.fr (J.-M.L.); 2 CIRAD, PHIM, F-34398 Montpellier, France; Denis.Filloux@Cirad.Fr (D.F.); emmanuel.fernandez@cirad.fr (E.F.); oumaima.moubset@cirad.fr (O.M.); charlotte.julian@cirad.fr (C.J.); laurence.blondin@cirad.fr (L.B.); philippe.roumagnac@cirad.fr (P.R.); 3 PHIM Plant Health Institute, University Montpellier, CIRAD, INRAE, Institut Agro, IRD, F-34398 Montpellier, France

**Keywords:** MinION, nanopore sequencing, rolling circle amplification, viral metagenomics, CRESS DNA, capulavirus, homopolymer

## Abstract

Next-generation sequencing (NGS), through the implementation of metagenomic protocols, has led to the discovery of thousands of new viruses in the last decade. Nevertheless, these protocols are still laborious and costly to implement, and the technique has not yet become routine for everyday virus characterization. Within the context of CRESS DNA virus studies, we implemented two alternative long-read NGS protocols, one that is agnostic to the sequence (without a priori knowledge of the viral genome) and the other that use specific primers to target a virus (with a priori). Agnostic and specific long read NGS-based assembled genomes of two capulavirus strains were compared to those obtained using the gold standard technique of Sanger sequencing. Both protocols allowed the detection and accurate full genome characterization of both strains. Globally, the assembled genomes were very similar (99.5–99.7% identity) to the Sanger sequences consensus, but differences in the homopolymeric tracks of these sequences indicated a specific lack of accuracy of the long reads NGS approach that has yet to be improved. Nevertheless, the use of the bench-top sequencer has proven to be a credible alternative in the context of CRESS DNA virus study and could offer a new range of applications not previously accessible.

## 1. Introduction

Recent advances in metagenomics applied to viruses has fostered a greater inventory of the viral diversity [1,2,3,4]. Hence, the large scale sampling of oceanic water [5,6], plants, animals, and humans [7,8,9,10], extreme environments [11], or the mining of genomic and transcritptomic data [12,13,14,15] have completely shifted our understanding of viral diversity and the function of viruses in host populations or even at the global ecosystem scale. However, these inventories remain largely incomplete, and the current knowledge of the virus diversity probably only represents the contour of the extant diversity [4]. The collection of hundreds of new genome sequences with, sometimes only remote resemblance to known viruses led to the acceptance of genome from metagenomic studies as genuine and legitimate taxonomic material for the description of new viruses [16,17,18,19,20,21], even without knowledge of the phenotype or the host associated to these viruses [17,22].

Yet, as access to metagenomics is usually costly and requires sophisticated technical expertise in data management and analysis [23,24]; for day-to-day analysis, classical Sanger sequencing remains more common [25]. Also, despite the potential of third generation sequencing technique to provide, in real time, hundreds of sequences with read length of more than 15 kb [26,27], the resulting assembled genomes remains tainted with doubt. Indeed, assembly of the low base quality reads is required to obtain more accurate contigs but may results in chimeric genomes, or miss less frequent variants [17,28,29]. It would thus be beneficial to evaluate the use of third generation sequencing as an alternative to the gold standard of cloning and Sanger sequencing for everyday virus characterization. Indeed, several studies have successfully employed third-generation sequencing for virus detection or full genome recovery. It has been largely used to detect and sequence full genomes of a range of animal and human viruses, including influenza [30], Ebola [31], Dengue [32], Zika [33], or SRAS-Cov-2 [34]. Recently the method was also successfully applied to plant viruses for some yam infecting viruses [29], maize yellow mosaic virus [35] and plum pox virus [36].

Here, within the context of the study of circular replication-associated protein encoding single stranded (CRESS) DNA viruses (from the *Cressdnaviricota* phylum), we harness the power of third-generation sequencing for routine full genome assembly of viruses. A breakthrough in CRESS DNA virus studies was associated with the development of protocols using isothermal rolling circle amplification (RCA) in frequent association with enzymatic restriction, cloning and Sanger sequencing [37]. Metagenomic protocols using RCA were soon developed and greatly improved our knowledge of the CRESS DNA virus diversity [38,39]. Yet, the use of Illumina based protocols remains laborious and expensive for day-to-day viral genome characterization and sequences analysis usually requires the use of complex bioinformatics tools. The field would thus benefit from the development of a more convenient protocol on the MinION platform from the Oxford Nanopore Technologies (ONT). Indeed, recent studies have paved the way towards using nanopore sequencing, either from direct sequencing of total DNA extracts or after the application of RCA, to achieve full genome sequencing of CRESS-DNA viruses in general [40,41,42] or viruses from the *Geminiviridae* family in particular [43,44,45].

Two alternative protocols for use on the MinION bench-top sequencing device were designed in this study and the assemblies of the nanopore-sequenced reads of multiple strains of capulaviruses infecting a *Medicago arborea* plant [46] were compared to the Sanger genome sequences. *Capulavirus* is one of the nine genera of the *Geminiviridae* family [47]. This family is composed of plant-infecting viruses with genomes comprising one or two circular ssDNA of 2.5–5.2 kb encapsidated in twinned icosahedral (geminate) particles. They are transmitted by a high range of hemipterans (whiteflies, leafhoppers, aphids, and treehoppers) [48,49]. Whereas members of the *Geminivirus* family were first described in 1993 (ICTV), the standardization of RCA based protocols lead to an explosion of its known diversity and the family regularly counts new genus-level lineage addition [47,50,51,52]. Following the description of the Euphorbia caput-medusae latent virus (EcmLV), the genus capulavirus has been proposed [53]. Their genome length ranged between 2.7 and 2.8 kb with two intergenic regions. The replication-associated protein (Rep) is expressed from a spliced complementary-strand transcript. A unique feature of capulavirus genomes is a complex arrangement of possible MP-encoding ORFs located in the 5′ direction from the coat protein gene (*cp*) [47]. It is known to be transmitted by only two species of aphids: *Aphis Craccivora* [49] and *Dysaphis plantaginea* [54].

Our analysis revealed that MinION sequencing followed by read assembly results in genome sequences mostly similar to the consensus of the virus population that was obtained after Sanger sequencing of multiple isolates. The two alternate protocols used, one that does not required knowledge of the viruses present within the sample (without a priori) and the other designed to specifically amplify a given virus (with a priori), were successful for full genome assembly of the two capulavirus strains. MinION assembled consensus sequences present with more differences in the homopolymeric tracks but remain closely related to any sequence of the sample than any Sanger sequence to one another. Overall, both nanopore-based protocols are adapted to the genome size of the CRESS DNA viruses and the cost of the method is on par with the Sanger sequencing approach.

## 2. Material and Methods

### 2.1. Sampling and DNA Extraction

Leaf samples of an apparently asymptomatic *Medicago arborea* were collected in November 2019 at Montferrier-sur-Lez (France). The sample was stored at −20 °C before use. Total DNA was extracted using the DNeasy Plant DNA extraction kit (Qiagen, Hilden, Germany), following the manufacturer’s instructions. DNA extract was stored at −20 °C before use. From a previous analysis [46], using a PCR amplification and Sanger sequencing, two strains of the capulavirus Trifolium virus 1 (TrV1-B and TrV1-C) were identified into the sample.

### 2.2. Full Genome Cloning and Sanger Sequencing

Pairs of abutting primers were designed to recover the full-length genome of TrV1-B and TrV1-C isolates. A two-step amplification was achieved, including a first amplification step using either the primer pair 3580F-CAPULUZARB-1F: 5′-ACT TGC CTG TCG CTC TAT CTT CTC CCT TGG AGA TGT AAT CTG CCA CGT CAG-3′, and PR2-CAPULUZARB-2R: 5′-TTT CTG TTG GTG CTG ATA TTG CGG AGT TTT TGA GGA ACG AGG AAT ACT TAG AGC TTC A-3′ for amplifying TrV1-B genomes or the primer pair 3580F-CAPUCORO-1F: 5′-ACT TGC CTG TCG CTC TAT CTT CAA CTG TCC TCC CTT TGC AAT GTA GTC AGC C-3′ and PR2-CAPUCORO-2R: 5′-TTT CTG TTG GTG CTG ATA TTG CCG AGG AGC GAG GAC TTC TTA AGG CAA GT-3′for amplifying TrV1-C genomes. Amplification was carried out using the GoTaq^®^ Master Mix Kit (Promega Corporation, Madison, WI, USA) and the following conditions: an initial denaturation at 95 °C for 5 min, 8 cycles at 94 °C for 30 s, 60 °C for 30 s, 72 °C for 3 min, and a final extension step at 72 °C for 10 min. A common second amplification step was then performed using the primer pair (3580F: 5′-ACT TGC CTG TCG CTC TAT CTT C-3′ and PR2: 5′-TTT CTG TTG GTG CTG ATA TTG C-3′) and the GoTaq^®^ Master Mix Kit (Promega Corporation, Madison, WI, USA). The amplification conditions were as followed: an initial denaturation at 95 °C for 5 min, 25 cycles at 94 °C for 30 s, 60 °C for 30 s, 72 °C for 3 min, and a final extension step at 72 °C for 10 min. Amplification products of approximately 2.7–2.8 kb were gel purified, ligated to pGEM-T (Promega, Madison, WI, USA) and sequenced by standard Sanger sequencing using a primer walking approach.

### 2.3. Minion Sequencing

Two alternative protocols for MinION sequencing were used. In the first protocol, called hereafter the RCA-MinION protocol, a RCA amplification was performed using *Phi*29 DNA polymerase (Illustra TempliPhi Amplification Kit, GE Healthcare, Chicago, IL, USA) by mixing 2 µL of total plant DNA extract with 5 µL of Sample Buffer before incubation at 95 °C during 3 min. After cooling at room temperature, 0.2 µL of enzyme mix and 5 µL of Reaction Buffer were added before incubation at 30 °C for 6 h followed by 20 min of polymerase deactivation at 65 °C. RCA products were cleaned-up using 2× of Sera-Mag Select Size Selection beads (GE Healthcare) and the 10 µL eluate were digested with 1 µL of T7 Endonuclease I (NEB), 2 µL of 5× buffer in a 10 µL reaction volume at 37 °C during 1 h. The fragments were purified with a 1× Sera-Mag Select Size Selection beads and eluate with 10 µL of purified water. Library construction for MinION sequencing was performed using the PCR Barcoding Kit (SQK-PBK004), following the manufacturer’s instructions but using SeraMag Select Size Selection beads (GE Healthcare) for DNA purification. Sequencing was performed as described below for the PCR-MinION procedure. Two Flongles (flow cell dongle) FLO-FLG001 were used for sequencing. Whereas in the first Flongle, a single RCA amplicon was sequenced, in the second, three distinct RCA amplicons were multiplexed.

In the second protocol, called hereafter the PCR-MinION protocol, a two-step amplification was carried out. In the first PCR, both sets of abutting primers (3580F-CAPULUZARB-1F/PR2-CAPULUZARB-2R and 3580F-CAPUCORO-1F/PR2-CAPUCORO-2R) described above for respectively amplifying the genomes of strains TrV1-B and TrV1-C and the GoTaq^®^ Master Mix Kit (Promega Corporation, Madison WI, USA) were employed. The amplification conditions were as follows: an initial denaturation at 95 °C for 5 min, 15 cycles at 94 °C for 30 s, 60 °C for 30 s, 72 °C for 3 min, and a final extension step at 72 °C for 10 min. The second amplification step was performed using the cDNA Primer (cPRM) supplied in the PCR-cDNA Sequencing Kit (SQK-PCS109) and the LongAmp Taq 2X Master Mix Kit (New England Biolabs, Evry, France). The amplification conditions were as followed: an initial denaturation at 95 °C for 30 s, 20 cycles at 95 °C for 15 s, 62 °C for 15 s, 65 °C for 3 min, and a final extension step at 65 °C for 6 min. The amplicons were purified using Agencourt AMPure XP beads (Beckman Coulter, Brea, CA, USA) and MinION sequencing library was constructed using the PCR-cDNA Sequencing Kit (SQK-PCS109), following manufacturer’s instructions. Sequencing was then performed on Flongle (FLO-FLG001) using MinKNOW software 19.06.8. Three Flongles were used, two for TrV1-B (Flongle 13 and Flongle 14) and another one for TrV1-C (Flongle 15).

### 2.4. MinION Sequence Assembly

For the reads obtained through the two MinION protocols, accurate basecalling was performed using Guppy (v4.09 or 4.015; [55]). Demultiplexing and adapter removal was then performed using Porechop v0.2.4 [56]. Quality of the reads was investigated using NanoPlot v1.33.0 [57].

**Figure 1:**
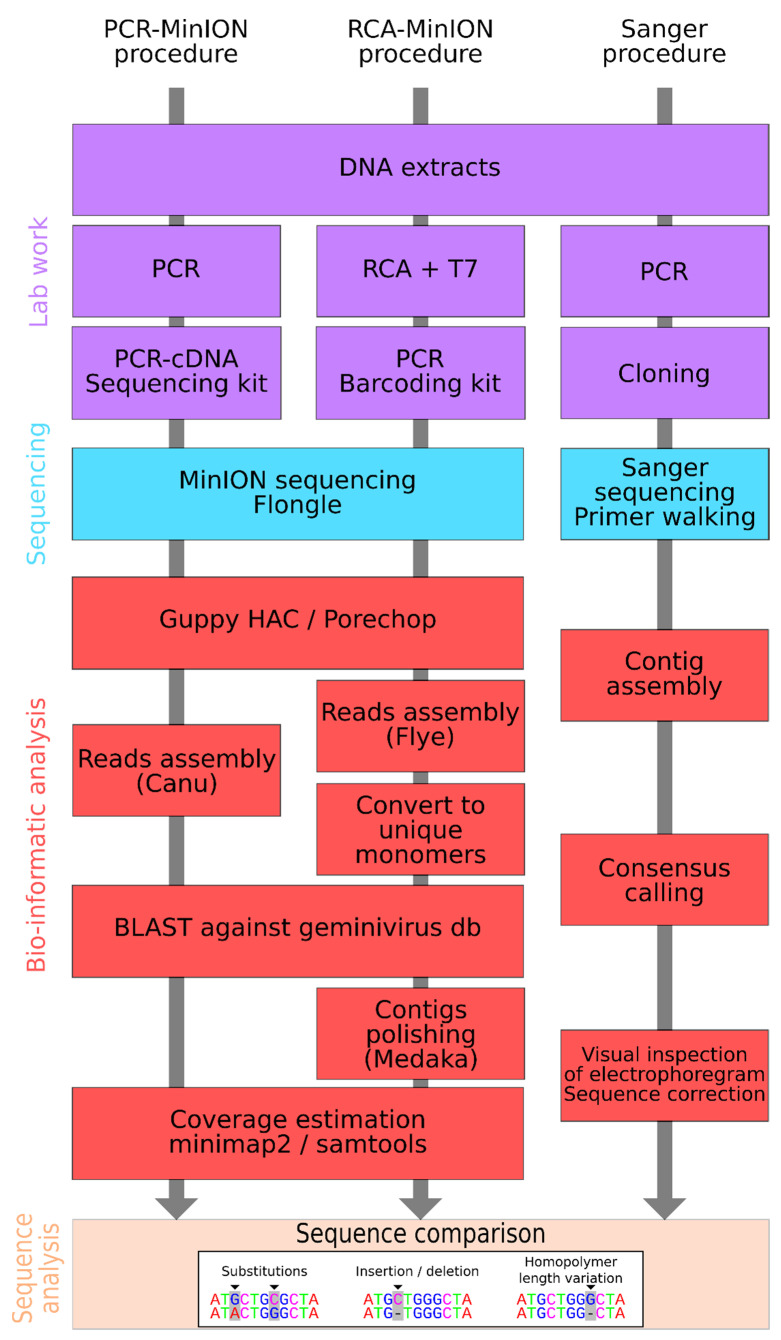
Schematic representation of the three distinct sequencing methodologies used including the molecular biology procedure (purple boxes) the sequencing procedure (light blue boxes), the bio-informatics procedure (red boxes) and the method comparison (orange boxes).

The demultiplexed reads obtained from RCA-MinION procedure were assembled with FLYE 2.6 [58], using the “meta” and “plasmid” parameters and when possible circularized as monomers using a homemade script. Contigs were then subjected to a BLASTn search against a CRESS DNA reference sequence database obtained from GenBank. CRESS DNA contigs were then polished using Medaka v1.2.2 [59]. PCR-MinION sequences higher than 1500 nt for one run (TrV1-C) and 2000 nt for the two other run (TrV1-B), were assembled using Canu v1.8 [60]. CRESS DNA contigs were filtered using BLAST as described above. Contigs coverage was estimated after mapping the raw reads back to the assembled CRESS DNA sequences using Minimap2 v2.17 [61] (Figure 1).

### 2.5. Sequence Comparison and Phylogenetic Analysis

All the available capulavirus full genome sequences were downloaded from GenBank on 4 March 2021 and aligned with the sequences of strains TrV1-B and TrV1-C obtained after Sanger sequencing using MAFFT v7.475 [62]. A maximum likelihood (ML) phylogenetic tree was inferred using FastTree2 [63] using the “gtr” and “gamma” parameters. Branch supports were tested using SH-like local supports. Tree edition was performed using the ape R package [64]. In order to properly classify the sequences obtained, an analysis that include a subset of representative capulavirus from GenBank and the Sanger sequence obtained in this study was performed using SDT1.2 [65].

Sequences obtained using the three distinct protocols (i.e., Sanger, PCR-MinION, and RCA-MinION procedures) were aligned together with MAFFT v7.475 before manual edit of the alignment. A home-made R script was used for sequence comparison and mutation count. Mutations were classified in three categories: substitution, insertion/deletion (INDEL) and homopolymer length variation (HLV) (Figure 1). ML trees were inferred from these alignments using FastTree2 as described above.

## 3. Results and Discussion

### 3.1. Sanger Sequences

A total of 46 sequences were obtained after cloning and Sanger sequencing. These sequence groups in two distinct clades (Figure S1) that share a mean identity of 90.3%. Within the first clade (*n* = 19), sequences present with identity ranging from 99.5 to 100% with each other and were most similar to the isolate BG2_capuz_47 of the B strain of TrV1 (GenBank accession number MW698819) with a minimum identity of 99.7%. Within the second clade (*n* = 27), sequences present identity ranging from 99.0 to 100% with each other and were most closely related to the isolate BG2_coro_02–2 of the C strain of TrV1 (GenBank accession number MW698820) with a minimum identity of 99.1%. Therefore, our two groups of sequences belong to two distinct strains (TrV1-B and TrV1-C) of the capulavirus *Trifolium virus 1* species (Figure S1). It is important to notice here that among the 46 isolates, five of the TrV1-B isolates and seven of the TrV1-C isolates present with defective genomes. Two of the TrV1-B defective isolates have deletions that encompass a fraction of the V3 gene, two other isolates have deletions within the gene of the replication-associated protein and one last has a deletion that encompasses the *rep* gene. Four sequences of the TrV1-C isolates have deletions that encompass a fraction of the CP gene. Among these, the deletions also span a fraction of the V3 gene or the *rep* gene, depending on the isolate. One isolate has a deletion that encompasses a fraction of all the gene encoded in the complement strand. These twelve defective sequences were excluded from downstream analysis. The genomic organization of the remaining sequences confirms the presence of a short intergenic region (SIR) and a long intergenic region (LIR), a characteristic inverted repeat potentially capable of forming a stem–loop structure that included a conserved nonanucleotide sequence TAATATTAC present at almost all geminivirus virion-strand replication origins, the *cp*, a spliced complementary-strand intron-containing transcript which expresses replication-associated protein gene (*rep*), a large complementary-sense ORF (C3) that is completely embedded within the *rep* gene, and a complex arrangement of possible MP-encoding ORFs located in the 5′ direction from the *cp* gene, which is a unique feature of *Capulavirus* genomes [47]. Four and three sequences of the TrV1-B and -C strains, present truncated ORFs. The full genome sequences of the isolate without ORFs truncation are available on GenBank under the accession numbers MW698819–MW698821 and MW768713–MW768736.

### 3.2. Long Read Sequencing and Assembly

**Figure 2:**
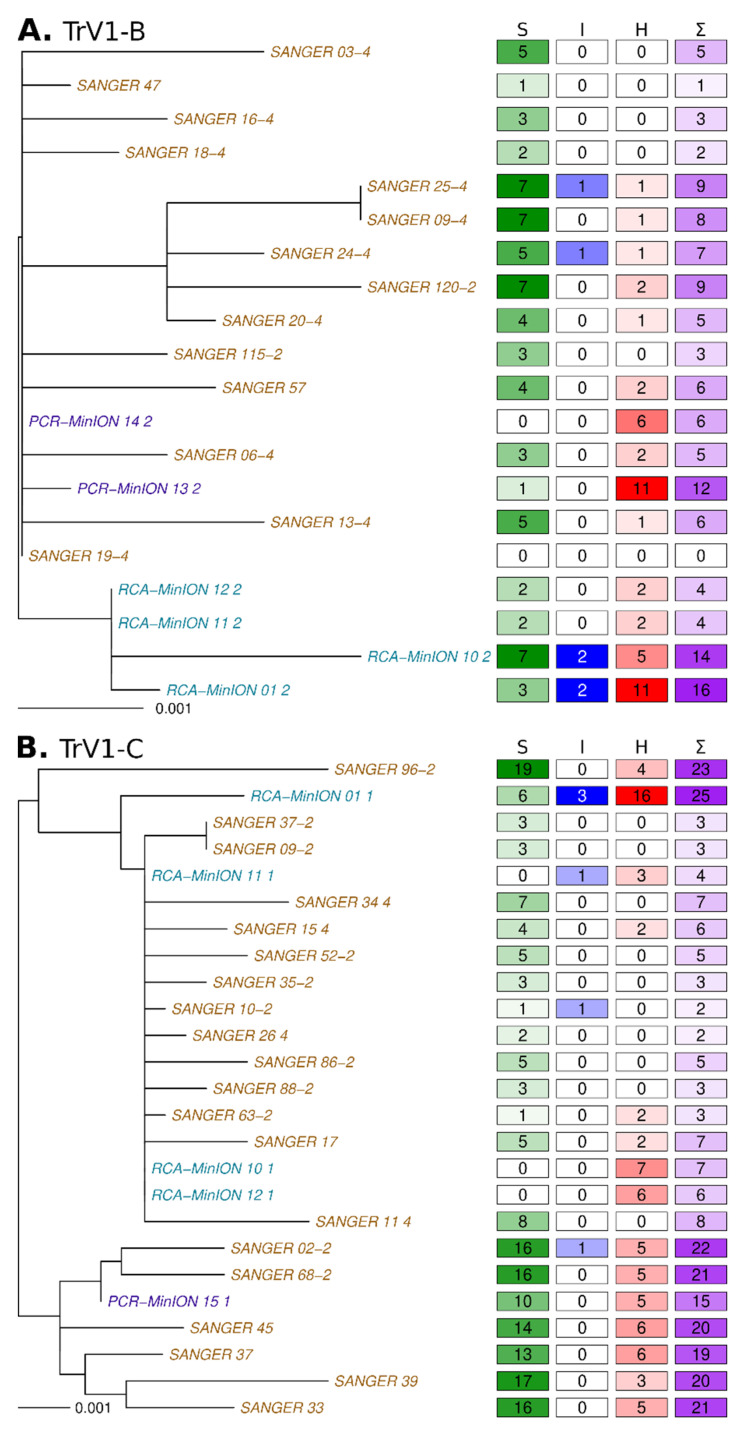
Maximum likelihood phylogenetic tree of all the sequences of the TrV1-B (**A**) and TrV1-C (**B**) strains obtained after Sanger sequencing (brown tips) or MinION sequencing followed by read assemblies (blue and purple tips for the RCA-MinION and PCR-MinION procedures respectively) on the left along with a matrix presenting the number of mutation relative to the Sanger consensus on the right. The numbers in the four columns present the substitutions (S), INDELs (I), HLVs (H) and the sum (Σ) of all these variations.

**Figure 3:**
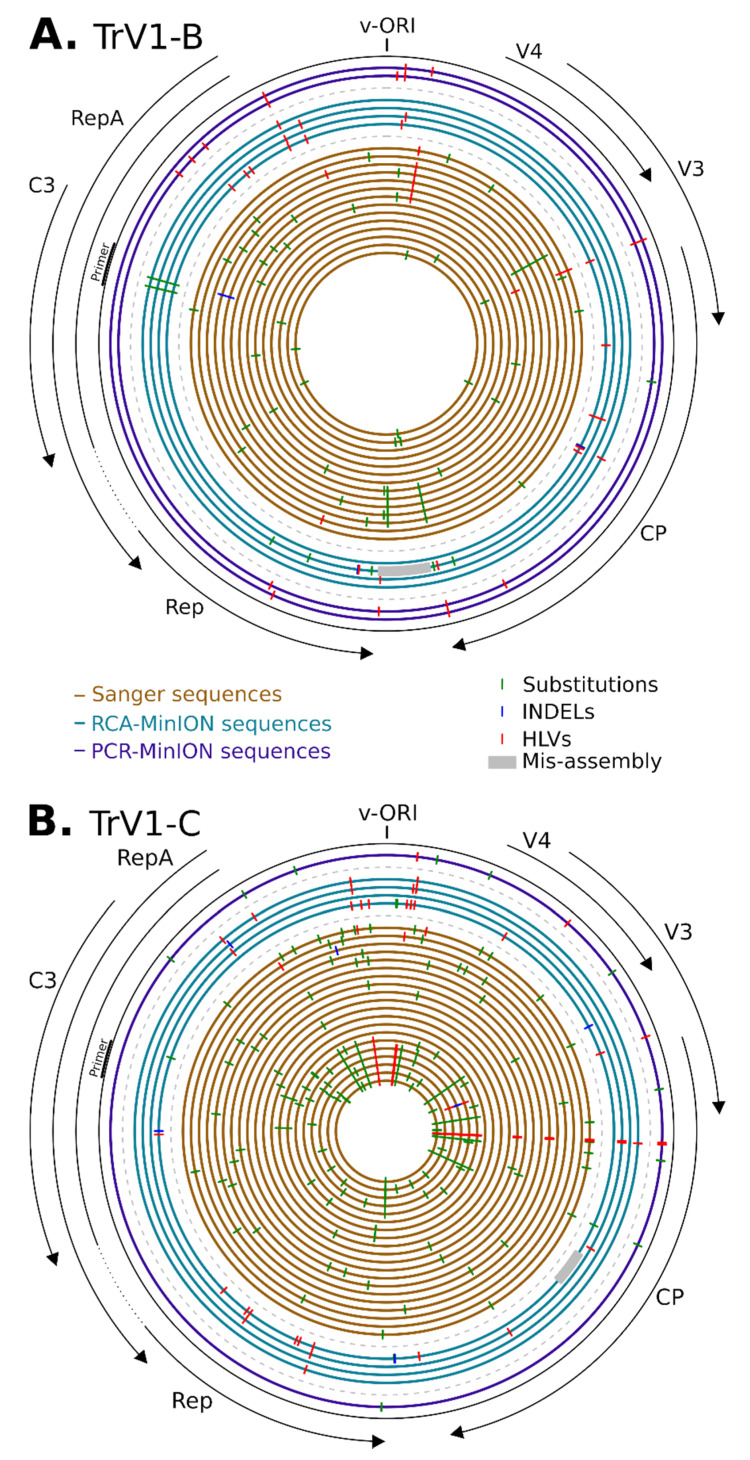
Diagram presenting the positions of the mutations along the genome of the TrV1-B (**A**) and TrV1-C (**B**) isolates and assemblies. Concentric circles represent each complete genome sequence obtained, with from the center to the outer, Sanger sequences in brown, RCA-MinION in blue and PCR-MinION in purple. Substitutions, INDELs and homopolymer length variations are represented with green, blue and red ticks respectively. Grey areas represent large deletions. Positions are relative to reference sequences MW698819 for TrV1-B and MW698820 for TrV1-C. The origin of replication (v-ORI) is indicated on top and the ORFs are represented on the outside of the figure.

The RCA-MinION generated reads that confirmed the presence of both TrV1-B and TrV1-C strains in the *Medicago arborea* sample (Figure 2). The raw sequencing statistics are available in Table 1. From Flongle 1 and Flongle 2, 188,123 and 273,088 raw reads were obtained from which 110,830 (59%) and 152,076 (56%) barcoded reads passed the quality control (Figure S2), respectively. The median read length was 1154 bp with a longest read of 10,491 bp. From Flongle 1, only 27 reads (0.02%) mapped with the capulavirus references. From Flongle 2, barcodes were retrieved from 65,413 reads, 40,242 reads and 46,421 reads for each of the three barcodes, respectively. From 6 to 8.5% of those reads mapped with the capulavirus sequences. Although it was performed on the same DNA extract, RCA amplification yielded more than two order of magnitude less viral sequence for the Flongle 1 amplification than those performed for Flongle 2. It highlights known bias of the RCA amplification [66,67] that were already evidenced in the context of CRESS virus amplification [68]. All the four distinct sequence sets (one barcode sample from Flongle 1 and three for Flongle 2) were then submitted to the assembly and circularization pipeline. For each of the four barcodes, unique contigs corresponding to the full genome sequences of the two strains were obtained. The four TrV1-B sequence lengths ranged from 2745 to 2769 bp and were at least 99.5% similar to any Sanger sequence. One of the TrV1-B sequences (RCA-Minion_10_2, Flongle 2 barcode 10, Table 1) present with a region that seems to be mis-assembled (100 nt in length, see grey tracks in Figure 3A). The four TrV1-C sequences length ranged from 2763 to 2771 bp and were at least 99.1% similar to any Sanger sequence. Again, a probable mis-assembly (68 nt in length, grey tracks in Figure 3B) was present within one sequence (RCA-Minion_01_1). As none of the raw minion reads supported the presence of this putative recombinant region, it has not been taken into consideration for further analyses.

The PCR-MinION generated reads that also confirmed the presence of TrV1-B and TrV1-C strains after RCA-MinION and Sanger sequencing. From Flongle 13, Flongle 14, and Flongle 15, 492,922, 414,665, and 768,144 raw reads were obtained from which 386,099 (78.3%), 371,700 (89.6%), and 714,730 (93.0%) reads passed the quality control (Table 1, Figure S2). The median length of the passed reads was 643 bp with a longest read of 9816 bp. From Flongle 13, 14 and 15, 380,933 reads (98.7%), 367,381 reads (98.8%) and 337,338 reads (47.2%) mapped with the TrV1-B and -C reference sequences. All the three read sets were then submitted to the assembly. For every Flongle, contigs corresponding to the full genome sequence of the TrV1-B and TrV1-C Sanger references were obtained. The two TrV1-B sequences length ranged from 2731 to 2739 bp and were at least 99.7% similar to any Sanger sequence. The TrV1-C sequence is 2754 bp length and were at least 99.3% similar to any Sanger sequence (Figure 2).

### 3.3. Sequences Comparison

In order to more precisely determine the nature of the differences between the sequences obtained through the different procedures, per capulavirus strains, all the full genome sequences obtained were compared to the consensus of the Sanger sequences. With more than 99.1% nucleotide identity for both the RCA-MinION and PCR-MinION sequences, the two methods demonstrate their ability to recover full genomes that are accurately assigned to strains TrV1-B and -C and whose sequence are representative of the viral population they originate. However, none of the assemblies obtained from MinION sequences was 100% identical to the Sanger sequence.

Beside mis-assemblies (grey tracks on Figure 3), the differences between sequences were classified in three categories with substitution (green ticks on Figure 3), INDEL (blue ticks) and HLV (red ticks). It must be noticed here that HLVs are a category of INDELs but were count separately as HLVs are recognized as the main source of errors in nanopore sequencing [69,70,71,72].

First, Sanger sequences differ from each other (and to the consensus) mostly with substitutions (accounting for 79.5% of the variations) and more marginally with HLVs (19.4% of the variations) and INDELs (1.5% of the variations) (Figure 2). Except for two of the TrV1-C sequences, all the other Sanger sequences were unique and the number of variations between all these sequences was up to 12 and 27 for TrV1-B and TrV1-C, respectively. A total of 46 and 113 polymorphic sites were present in the Sanger sequences for TrV1-B and TrV1-C, respectively (Figure 3). Whereas these polymorphic sites tend be more frequently present within non-coding regions (binomial test *p*-value of 0.090 and 0.016 for TrV1-B and TrV1-C, respectively), these sites had globally more mutations among all the Sanger sequences (binomial test *p*-value of 3.7 × 10^−3^ and 7.7 × 10^−6^ for TrV1-B and TrV1-C, respectively). Whereas sequencing errors can explain a fraction of the mutation, with the high accuracy of Sanger sequencing in mind (per base Phred quality score of 50 [73]), one can expect that most of the variations uncovered during the analysis are genuine and represent the biological variation associated with the diversity of the viral population infecting the plant [74,75,76].

**Figure 4:**
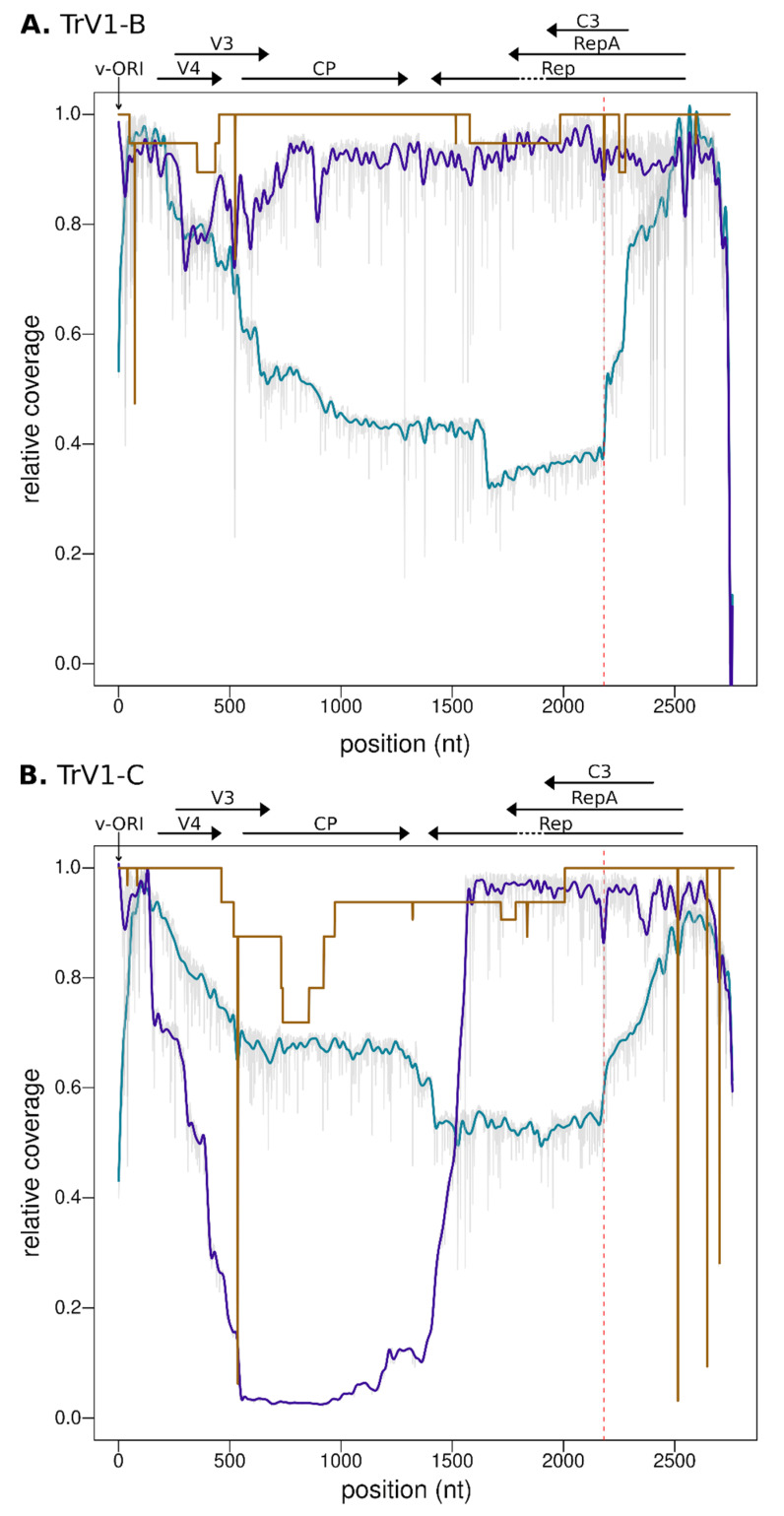
Coverage plot along the reference genome of TrV1-B (**A**) and TrV1-C (**B**). The coverage, interpreted here as the fold coverage of a given position divided by the maximum coverage of the whole genome, is represented with a brown line for Sanger sequences, a blue line for RCA-MinION sequences and a purple line for PCR-MinION sequences. MinION coverage are interpolations of the raw coverages presented here in grey on the background. The position of the abutting primers used in the PCR-MinION procedure is indicated by the vertical red dotted line. Positions are relative to the reference genome MW698819 for TrV1-B and MW698820 for TrV1-C. ORFs of these reference genomes are symbolized with horizontal arrows on top of the figure. The Origin of replication is indicated with an arrow. The position 1 of each sequence correspond to the nick-site within the stem loop structure.

The analysis of the RCA-MinION and PCR-MinION sequences revealed a different pattern of polymorphism. Two sequences, one of TrV1-B and one of TrV1-C, both assembled from a restricted number of reads (Flongle 1, RCA-MinION_01-1 and RCA-MinION_01-2) were, as expected, less accurate. Coverage of the assembly, (e.g., representing the mean number of times every position of an assembly was read) is therefore a good indicator of the reliability of the resulting assembly (Figure 4). Among the nine other MinION sequences assembled, seven present with no substitution to the consensus and one with ten substitutions (Figure 2). Through the TrV1-B sequences there was few common mutations: only a single HLV was common between Sanger and MinION assemblies (Figure 3A). PCR-MinION and RCA-MinION sequences presented four common HLVs (Figure 3A). For TrV1-C, Sanger sequences presented one substitution, no INDEL and three HLVs in common with MinION sequences (Figure 3B). PCR-MinION and RCA-MinION sequences presented one common substitution, three common HLVs and no common INDELs (Figure 3B). As these sequences were obtained after the assembly of reads, we were unable to catch the diversity of the distinct variants forming the viral population but rather to obtain a sequence very similar to the consensus of that population. Conversely to the reduced number of substitutions in comparison to the consensus, the assemblies present with larger numbers of INDELs (from 1 to 3) and HLVs (2 to 16). Some of these variations were also found in the Sanger sequences and it is probable that the assemblies actually represented some of the variability within the population. Nevertheless, for six INDELs and 38 HLVs, no corresponding mutations were found, and most would induce frameshift or protein truncation (Figure 3). Despite the use of a dedicated sequence correction program, multiple HLVs remained in the assembled sequences.

### 3.4. Defective Genome and Sequence Coverage

Defective genomes are frequently detected within geminivirus populations [77]. For instance, twelve of the Sanger sequences displayed large deletions in comparison to full reference genomes. As MinION raw sequences are obtained after direct reads of either PCR or RCA amplicons, they should capture the diversity of defective genomes of the viral populations. Indeed, to obtain full genome assemblies using the PCR-MinION procedure, a selection of the reads approaching full genome size was required. The analysis of coverage of the reads (Figure 4) confirms the pervasive nature of defective genomes for both the TrV1-B and -C strains. The highest coverage was obtained for regions encompassing the stem-loop and reads were frequently missing most of the coding regions (see the blue lines for RCA-Minion). Importantly, it must be noticed here that the coverage inferred using the PCR-MinION procedure are not representative of the global population but rather represent the subset of virus that contains the priming site of the abutting primers used in the PCR (indicated with the verticals red dotted lines in Figure 4). The results indicate that every position of the TrV1-B strains present with a high coverage, most of the amplicons of TrV1-C were defective for a region encompassing the whole CP gene (from position 147 to 1559).

## 4. Conclusions

From an asymptomatic sample of *Medicago arborea*, two distinct strains of the capulavirus TrV1 have been cloned and sequenced by the Sanger methodology. Using both the *a priori* and the agnostic nanopore-based procedures, both TrV1-B and TrV1-C strains were detected, and full genome sequences were assembled. Despite being very similar to the consensus of Sanger sequences, mutations specific to the MinION assemblies were detected mostly within homopolymeric regions of the genomes, which is in agreement with other studies that have also pinpointed higher number of errors associated with homopolymer lengths [78,79]. Whereas it could be argued that the HLV errors would be reduced with the increasing accuracy of sequencing, the development of a new base caller technologies and correction algorithm [80], current MinION sequences assemblies may be avoided for some specific applications were the exact nucleotide sequence is required. Otherwise, for other applications, such as virus discovery, virus classification, or recombination analysis, MinION assemblies represent a competitive alternative to Sanger sequencing. Although, similarly to other NGS protocols, MinION based studies require the use of sophisticated bioinformatics tools for data management and analysis and despite the specific drawbacks on sequence quality, bench top sequencer such as the MinION would probably become routinely used in the laboratory. It allows a real-time detection and diagnostic of multiple viral strains or virus species in a single run. For niche applications, such as the exploration of geminivirus genomes, which do not exceed 10 kb, nanopore sequencing is poised to push the cost and performance limits of sequencing technologies. The high reactivity offered by the platform would make it more and more democratized as a mobile real-time plant disease diagnostic tool.

## Figures and Tables

**Table 1:** Long read sequencing statistics.

Flongle Id	Raw Reads	Passed Reads	Barcode ID	Trimmed Reads	Capulavirus Reads	TrV1-B Reads	TrV1-C Reads	Length TrV1-B Assembly	Length TrV1-C Assembly
1	188,123	162,263	1	110,830	27	14	13	2745	2771
2	273,088	215,143	10	65,413	4809	3230	1579	2769	2764
			11	40,242	3423	2244	1179	2748	2763
			12	46,421	2803	1585	1218	2746	2765
13	492,922	386,099	-	385,029	380,933	380,933	-	2731	-
14	414,665	371,700	-	370,755	367,381	367,381	-	2739	-
15	768,144	714,730	-	602,579	337,338	-	337,338	-	2754

## Data Availability

Publicly available datasets were analyzed in this study. Sanger sequences accession numbers [MW698819–MW698821, MW768713–MW768736] are available on NCBI Genbank. MinION data are available at the NCBI Short read archive under the BioProject [PRJNA715304].

## Supplementary Materials

The following are available online at https://www.mdpi.com/article/10.3390/microorganisms9050903/s1. Figure S1: Maximum likelihood phylogenetic tree of all the *Capulavirus* genus full genome sequences obtained from NCBI along with the two TrV1-B and TrV1-C genotypes obtained in this study; Supplementary Figure S2: Reads length (*x*-axis) against quality score (*y*-axis) for all passed reads (A and C) and capulavirus only passed reads (B and D) obtained using the RCA-MinION and PCR-MinION procedures.
