# Supplementary figures and images for: Nanopore Sequencing Is a Credible Alternative to Recover Complete Genomes of Geminiviruses
Source: Microorganisms. 2021 Apr 23;9(5):903. doi: 10.3390/microorganisms9050903 (PMC8147096; doi:10.3390/microorganisms9050903)

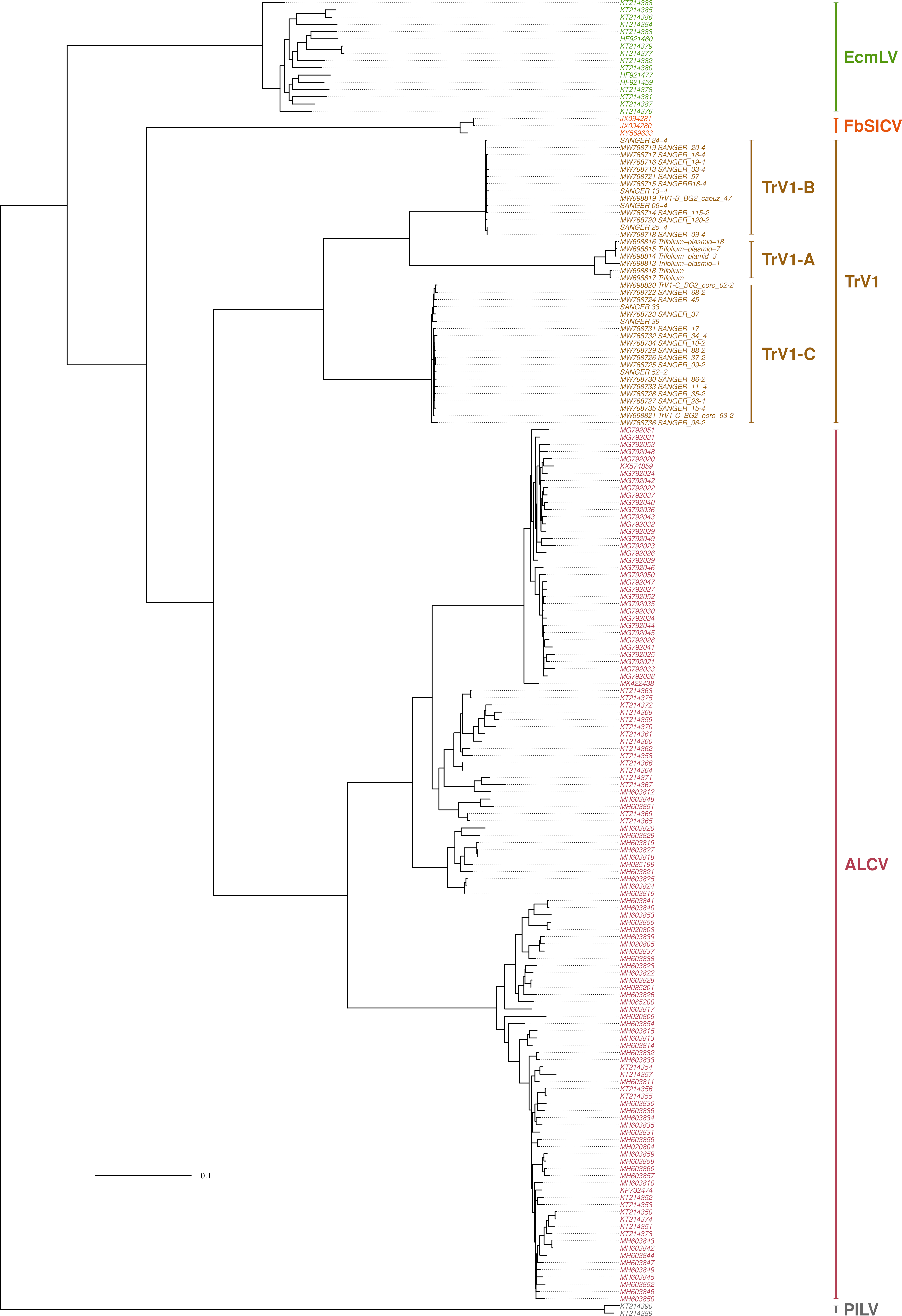

Supplement: Supplementary file 1 [file microorganisms-09-00903-s001.zip › SuppFigure1_arbrecapula.tiff]

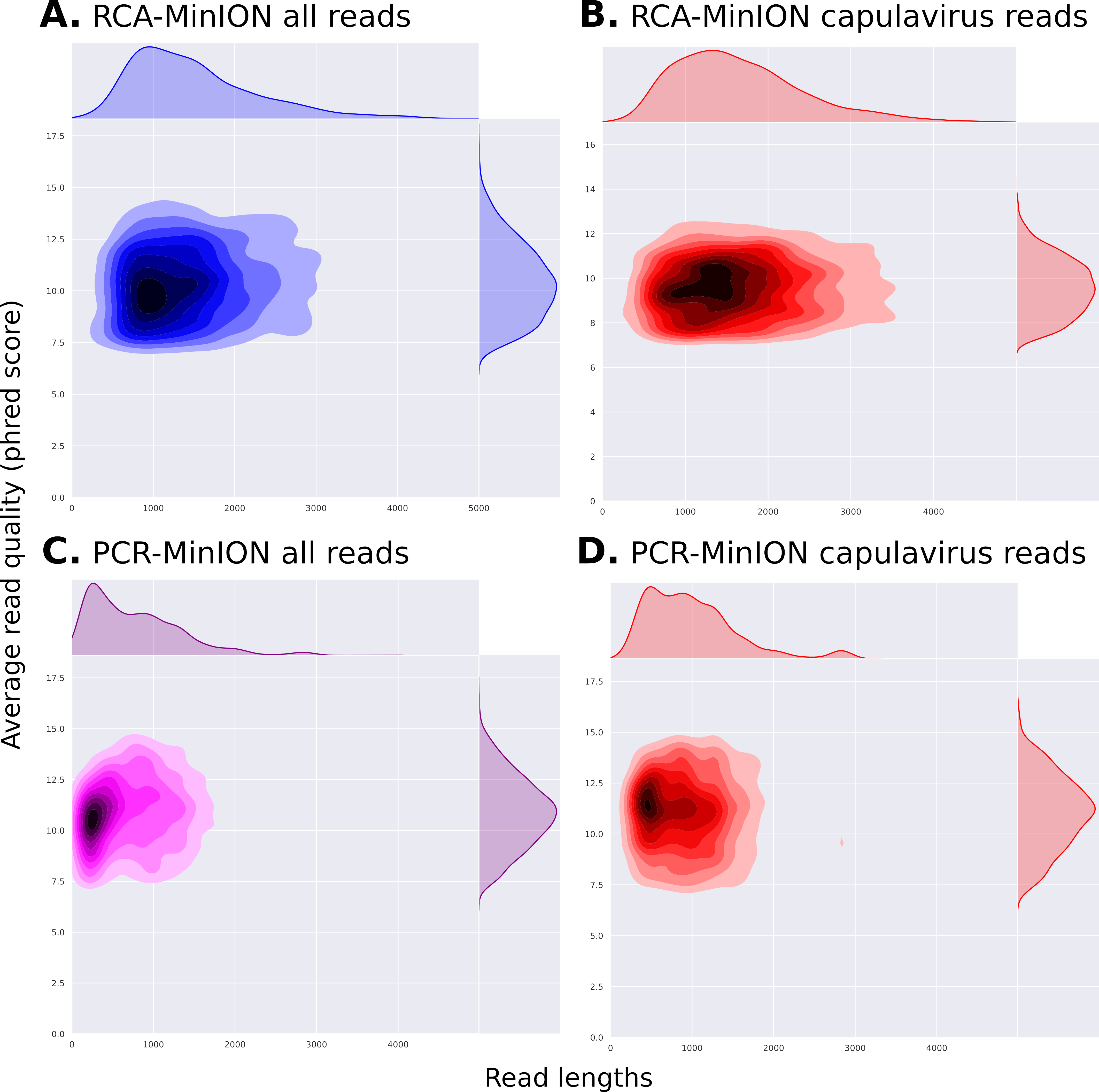

Supplement: Supplementary file 1 [file microorganisms-09-00903-s001.zip › SuppFigure2_quality_vs_length.tiff]
